# Evaluation of pathogen P21 protein as a potential modulator of the protective immunity induced by *Trypanosoma cruzi* attenuated parasites

**DOI:** 10.1590/0074-02760180571

**Published:** 2019-05-20

**Authors:** Cecilia Pérez Brandán, Andrea C Mesias, Leonardo Acuña, Thaise Lara Teixeira, Claudio Vieira da Silva

**Affiliations:** 1 Universidad Nacional de Salta, Instituto de Patología Experimental, Consejo Nacional de Investigaciones Científicas y Técnicas, Salta- Capital, Argentina; 2 Universidade Federal de Uberlândia, Instituto de Ciências Biomédicas, Uberlândia, MG, Brasil

**Keywords:** Trypanosoma cruzi, Chagas disease, attenuated vaccine, P21

## Abstract

**BACKGROUND:**

TcP21 is a ubiquitous secreted protein of *Trypanosoma cruzi* and its recombinant form (rP21) promotes parasite cell invasion and acts as a phagocytosis inducer by activating actin polymerisation in the host cell.

**OBJECTIVE:**

Our goal was to evaluate if the additional supplementation of rP21 during a prime/boost/challenge scheme with *T. cruzi* TCC attenuated parasites could modify the well-known protective behavior conferred by these parasites.

**METHODS:**

The humoral immune response was evaluated through the assessment of total anti-*T. cruzi* antibodies as well as IgG subtypes. IFN-γ, TNF-α and IL-10 were measured in supernatants of splenic cells stimulated with total parasite homogenate or rP21.

**FINDINGS:**

Our results demonstrated that, when comparing TCC+rP21 *vs.* TCC vaccinated animals, the levels of IFN-γ were significantly higher in the former group, while the levels of IL-10 and TNF-α were significantly lower. Further, the measurement of parasite load after lethal challenge showed an exacerbated infection and parasite load in heart and skeletal muscle after pre-treatment with rP21, suggesting the important role of this protein during parasite natural invasion process.

**MAIN CONCLUSION:**

Our results demonstrated that rP21 may have adjuvant capacity able to modify the cytokine immune profile elicited by attenuated parasites.

For many parasitic diseases, vaccines had the potential to overcome the lack of an efficient chemotherapeutic treatment ― or its limited efficacy ― and its associated side effects. There has been a great progress related to vaccine development for several parasitic diseases but for Chagas disease, as well as for other neglected diseases, the progress has not been so encouraging. However, in the past decade an increasing number of evidence suggests that a vaccine against *Trypanosoma cruzi* would be feasible and reasonable in terms of economic values. Several immunoprophylactic as well as immunotherapeutic attempts have been made in order to prevent *T. cruzi* establishment and persistence in the host. Most assessed protocols included heterologous, prime-boost immunisation schemes based on the combination of DNA vaccines and recombinant proteins.^1^ These were delivered or supplemented in various forms, including immune response enhancers such as adenoviruses,^2^ attenuated *Salmonella* sp,^3^ Modified Vaccinia Ankara^4^ and most recently, nanoparticles.^5^ Also, the use of new generation adjuvants based on saponin^6^ or Toll Like Receptors (TLR) agonists^7^ for instance has gained reawakened attention. In addition, the generation of genetically modified *T. cruzi* mutant strains and their use as experimental immunogens has shown a significant protection against lethal infection,^8^ ^,^ ^9^ and presumably no persistence in the host, rendering this alternative worth to keep working on.^10^ ^,^ ^11^ On the other hand, several parasite antigens, such us proteins from the trans-sialidase (TS) superfamily, or proteins *Tc*24 and *Tc*52 for instance, have also been tested in animal models.^12^

Among *T. cruzi* proteins, there is one that particularly gained our attention. P21 is a key protein secreted by all developmental stages of *T. cruzi.* It is mainly involved in cell invasion and modulation of the host immune response, although its role during parasite infection is not fully elucidated yet. It has been demonstrated that recombinant P21 (rP21), produced by heterologous expression, promotes *T. cruzi* cell invasion by confining to the chemokine receptor CXCR4 expressed in macrophages and acts as a phagocytosis inducer by activating actin polymerisation in the host cell.^13^ ^,^ ^14^ It has also been shown that rP21 participates in macrophages activation by increasing the production of myeloperoxidase, therefore recruiting immune cells such as lymphocytes and neutrophils. Even more, a decrease in blood vessel formation in the presence of rP21 has been observed leading to the hypothesis that its anti-angiogenic effect may be related to vascular abnormalities and to focal ischemia observed in chronic chagasic cardiomyopathy.^15^ ^,^ ^16^

In our experience and from others as well, a subpatent infection with naturally or genetically modified attenuated parasites is enough to confer strong protection against a subsequent infection.^10^ ^,^ ^17^ ^,^ ^18^ Hence, if rP21 is described as a determinant protein favoring parasite cell invasion and permanence in the host, we could speculate that the additional supplementation of rP21 during the immunisation process with the attenuated parasites would promote the continuity of the parasites in the host for a longer period of time, thus giving additional time for the development of a prophylactic immune response able to avoid a second parasite establishment. On the other hand, we would be also favoring the recruitment of immune cells in a larger extent being able to improve the protective immune response conferred by the attenuated parasites.

## MATERIALS AND METHODS

*Ethics statement* - All animal protocols adhered to the National Institutes of Health (NIH) ‘‘Guide for the care and use of laboratory animals’’ and were approved by the School of Health Sciences and by the Ethical Committee of the National University of Salta, Argentina (Nº 311/18).

*Protein and parasites preparation* - For the immunisation assays, recombinant *T. cruzi* protein rP21 was used as well as metacyclic trypomastigotes from the *T. cruzi* naturally attenuated TCC strain. rP21 was obtained from bacteria culture as previously described.^19^ Epimastigotes were grown at 28ºC in liver digested neutralised tryptose medium (LDNT), supplemented with 10% foetal bovine serum (FBS) until they reached log-phase. Metacyclics trypomastigotes were then obtained by epimastigotes differentiation under chemically defined conditions (TAU 3AAG medium: 190 mM NaCl, 17 mM KCl, 2 mM CaCl_2_, 8 mM phosphate buffer, 2 mM MgCl_2_, pH 6.8, supplemented with 0.035% NaHCO_3_, 10 mM l-proline, 50 mM sodium glutamate, 2 mM l-aspartate y 10 mM glucose) and harvesting differentiated parasites after 10 days. Complement resistant forms were purified by incubating differentiated parasites with normal human non decomplemented serum at 37ºC during 5 h. Purified parasites were then washed with PBS 1X twice, quantified under light microscopy and further used to inoculate experimental animals.

*Mice* - Six week old male C57BL/6 mice were used. The animals were obtained from the animal facility core of the School of Health Sciences of the National University of Salta, Argentina.

*Immunisation protocol* - Five mice per group (n = 5) were used throughout this study. Mice were maintained under standard conditions on 12 h light-dark cycle in a temperature controlled setting (25ºC) with food and water *ad libitum*. Mice under anesthesia were immunised intramuscularly (IM) with two doses, delivered at 4-week intervals, of 10^5^ metacyclic attenuated TCC parasites in combination with 20 µg of rP21. Control groups, including rP21 and attenuated parasites separately, as well as PBS-5% saponin (control -) were included all throughout the experiments. All the formulations were emulsified in PBS-5% saponin as a vehicle (Cat. Nº SAE0073-Sigma-Aldrich) in 50 µL final volume *per* mouse. Before challenge, blood was collected from all mice in order to collect sera samples for IgGs determination. Also, two mice from each group were sacrificed by CO_2_ exposure for spleen removal and further splenocytes isolation and cytokines measurements. The whole experimental protocol was repeated twice from immunisation to challenge.

*Specific antibody determinations* - Total IgG antibodies and IgG subtypes against *T. cruzi* and rP21were measured by the enzyme-linked immunosorbent assay (ELISA) using *T. cruzi* epimastigote homogenate (EH) by seeding 1 µg EH/100µl-Coating Buffer/well, and 0.5 µg rP21/100 µL-Coating Buffer/well, as antigens. To identify total antibodies and subtypes, plates were blocked with PBS-5% non-fat dry milk, and then incubated with sera samples (1:100 dilution, 100-µL/well) for 1 h. After washing three times, biotin-conjugated goat anti-mouse IgG (Cat. Nº B6649 - Sigma-Aldrich), IgG1 (Cat. Nº 550331 BD Biosciencies) and IgG2a/c (Cat. Nº 550332 - BD Biosciencies) in a 1:6000 dilution were added (100-µL/well). After 1 h incubation, the plate was washed three times with PBS and streptavidin-horseradish peroxidase conjugate was added (Cat. Nº 550946 - BD Biosciencies) and let for 1 h at 37ºC. Colour was developed with TMB Substrate Reagent Set (Cat. Nº 555214-BD Biosciencies) and monitored at 450 nm using a Tecan Infinite Pro microplate reader.

*Cytokine response* - Spleens were removed from euthanised immunised mice, macerated on a sterile mesh, and cells were resuspended in RPMI 1640 medium (Cat. Nº R7755 - Sigma-Aldrich). Following centrifugation at 160 × *g* for 10 min at 4ºC, the cells were resuspended in a lysis solution (0.17 M Tris pH 7.2, 0.16 M NH_4_Cl) to remove erythrocytes. The remaining splenocytes were washed three times with RPMI and resuspended in RPMI supplemented with 20 mM glutamine, 10% NaHCO_3_ and 10% FBS. Viability of cells was assessed by Trypan blue exclusion and cell number was determined in a Neubauer chamber. Splenocytes (10^6^cells/well, in triplicate) were cultured for 48 h at 37ºC and 5% CO_2_, with or without stimulation with 25 µg/mL rP21, 25 µg/mL of *T. cruzi* epimastigote homogenate (EH) or 50 µg/mL of phytohaemagglutinin (PHA). Cell culture medium was then collected to measure the released of IL-10, TNF-α and IFN-γ by ELISA according to the instructions of the manufacturer (optEIA enzyme-linked immunosorbent assay kits, BD-Biosciencies, San Diego, CA).

*Challenge infection in mice and parasitaemia* - To evaluate the response to *T. cruzi* upon immunisation, mice from each group were challenged with *T. cruzi* Tulahuen strain (500 blood trypomastigotes/mouse, intraperitoneally). Mortality was twice a week until 30 days post challenge. Parasitaemia was carried out according to previously standardised methods. Briefly, blood (10 µL) was drawn from the tail tip of mice under slight anesthesia, and the number of parasites *per* 100 fields was recorded from fresh blood mounts under bright-field microscope.

*Parasite DNA detection* - Thirty days after challenge mice were sacrificed by CO_2_ exposure to remove target organs. Total DNA from skeletal muscle and heart tissue (50 mg) was isolated using the ADN-Puriprep Highway nucleic acid kit (Cat. Nº K1205-250; InbioHighway), according to instructions provided by the manufacturer. Total DNA (10 ng per sample) was used as template, and real-time polymerase chain reaction (PCR) performed on a LineGene 9640 Sequence Detection System (BIOER) with Eva Green Supermix (Cat. Nº 92005; Bioline) and Tc18S rDNA-specific oligonucleotides (SAT_F 5’GCAGTCGGCKGATCGTTTTCG-3’ and SAT_R 5’TTCAGRGTTGTTTGGTGTCCAGTG-3’). Data was normalised to murine TNF-α (TNF_F 5’-TCCCTCTCATCAGTTCTATGGCCCA-3’ and TNF_R 5’CAGCAAGCATCTATGCACTTAGACCCC-3’).^20^

*Statistical analysis* - Statistical analysis was performed using the software GraphPad Prism V5.00. Continuous variables, such as antibody levels and parasite concentrations in blood samples, were analysed with the two-tailed Wilcoxon signed-rank test for time course plots and with the Mann-Whitney or Kruskal-Wallis test for single-day measurements with Dunn’s Multiple Comparison Test. Values are expressed as mean with standard errors of the mean (SEM) from at least two independent experiments, with three lectures per sample in each case. Differences between two groups considered significant are denoted as *p ˂ 0.05, **p ˂ 0.01, ***p ˂ 0.001 or ns no significant.

## RESULTS AND DISCUSSION

A few years ago we described a novel *T. cruzi* protein, named P21. We know that rP21 promotes *T. cruzi* cell invasion,^13^ therefore invading parasites could freely multiply inside the host cell and invade other cells. Besides, rP21 could also be acting as a phagocytosis inducer by activating actin polymerisation in the host cell ^14^ hence, in this scenario, parasites could be confined within the invaded cell due to this phenomenon. Consequently, and with these ideas in mind, we wonder what would be the role of rP21 in a prime-boost scheme based on highly attenuated parasites formulated with rP21, followed by a lethal challenge with virulent *T. cruzi* parasites.

**Fig. 1:**
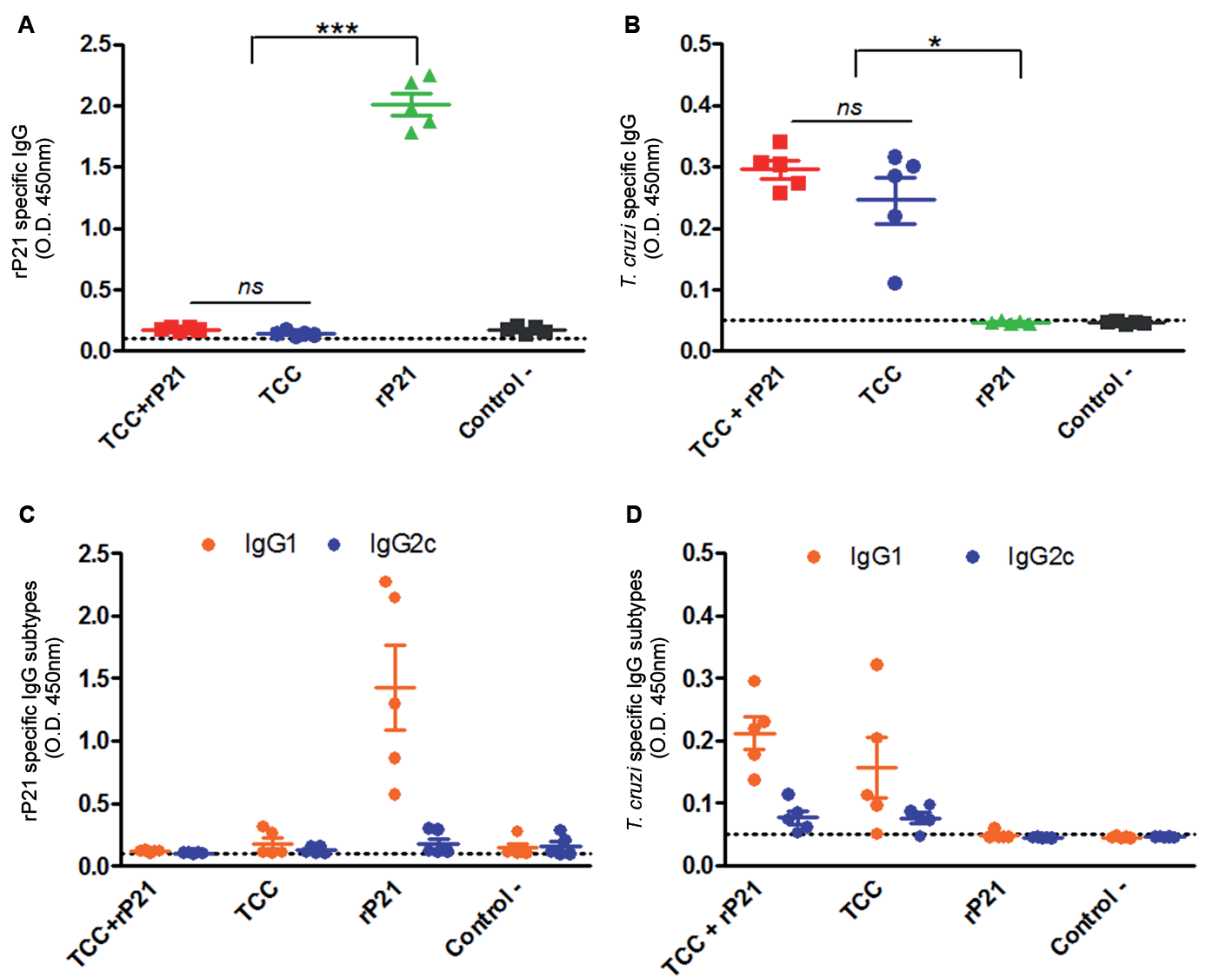
antibody response in immunised animals. Groups of mice (n = 5 per group) were immunised with TCC + rP21, TCC, rP21 and as a negative control, PBS- saponin 5% , which is the vehicle in which all the formulations were resuspended. Four weeks after the last immunisation blood was collected and the levels of total anti-rP21 (A) and anti-*Trypanosoma cruzi* IgGs (B) were measured in serum samples. Total anti-rP21 IgG1/IgG2c (C) and anti-*T. cruzi* IgG1/IgG2c (D) in serum samples was also measured. Data are presented as mean ± SEM. *, *** stand for p ˂ 0.05 and p ˂ 0.001 respectively, *ns* for no significant.

*Anti-rP21 specific humoral response is abrogated under the presence of attenuated parasites* - As a first instance we focused on determining the levels of total IgGs antibodies in sera from immunised animals. For this, mice were injected with two doses of TCC+rP21 emulsified in PBS-5% saponin with the corresponding control groups. Four weeks after the last immunisation, measurement of anti-rP21 specific antibody levels indicated that mice receiving a formulation based on TCC+rP21 were unable to maintain the levels of anti-rP21 IgGs elicited by the administration of rP21 alone (Fig. 1A). When total IgG levels were measured against a whole *T. cruzi* homogenate, the antibody response induced by the attenuated parasites is maintained despite the addition of rP21 (Fig. 1B) indicating that at least at this level the humoral total anti *T. cruzi* response is not affected.

We could speculate that the lack of detection of anti-rP21 specific antibodies in the TCC+rP21 group could be attributed to the endogenous production of P21 by the immunising parasites and further sequester of these anti-P21specific antibodies. Another possible explanation for the observed results could be related to the presence of a highly variable plethora of antigens when attenuated parasites are introduced. In this situation the addition of exogenous rP21 could be irrelevant, and lead to the generation of antibodies limited to more competitive antigens exposed by parasites.

Total *T. cruzi* and rP21 specific IgG1 and IgG2c antibody levels were measured to estimate the elicited T cell profile in immunised animals. TCC immunised animals are characterised for presenting a Th2 response based mainly in the production of *T. cruzi* specific IgG1 antibodies. In the case in which TCC parasites were formulated with rP21 (TCC+rP21) the response is maintained invariable. Neither anti-rP21 IgG1 nor IgG2c were detected in TCC+rP21 immunised animals (Fig. 1C). It has been shown that several *T. cruzi* recombinant proteins are able to generated Th1-driven antibodies in vaccinated mice;^21^ ^,^ ^22^ ^,^ ^23^ however, elevated levels of anti-rP21 IgG1 were detected in rP21 primed animals indicating that a Th1 bias profile for a *T. cruzi* recombinant protein is not always the rule. The Th2 profile found in TCC+rP21 immunised animals, with a predominance of anti-*T. cruzi* IgG1 antibodies and a lower proportion of anti-*T. cruzi* IgG2c antibodies, was similar to the one obtained for TCC primed animals, indicating that the addition of rP21 did not alter this profile (Fig. 1D). Thus, these results allow us to conclude that rP21 does not have the potential to modify the antibody profile triggered by TCC attenuated parasites. Still, we have evidenced that modulation of host immune response during TCC attenuated infection is possible. We have previously shown that administration of an eukaryotic expression plasmid encoding for murine IFN-γ could have beneficial effects such us increasing host specific antibody production in response to *T. cruzi* attenuated infection.^24^

**Fig. 2:**
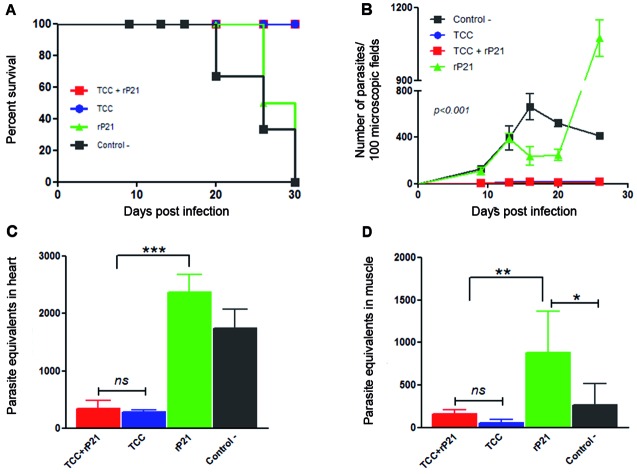
parasite burden in animals immunised with rP21 formulations after lethal challenge. Immunised mice as well as control group were infected with a lethal dose of *Trypanosoma cruzi* trypomastigotes of the Tulahuen strain of *T. cruzi* four weeks after the last immunisation. Survival was recorded twice a week until day 30 (A). Parasitaemia (B) was recorded twice a week until control group died and cardiac (C) and skeletal muscle parasite burden (D) was measured at the end of this period. *: p ˂ 0.05; **: p ˂ 0.01; ***: p ˂ 0.001 or *ns* no significant.

*Parasite load in peripheral blood and target organs after virulent challenge is not reduced by the presence of rP21* - The naturally attenuated TCC strain is well known for conferring strong protection against a later virulent infection. To evaluate the possible adjuvant capacity of rP21, all immunised mice were challenge with a lethal dose of *T. cruzi* virulent parasites, from Tulahuen strain (Tul), four weeks after the last immunisation. Survival after challenge was controlled twice a week (Fig. 2A), and showed that control groups, inoculated with PBS-5% saponin or rP21, died between 20 and 30 days after virulent infection, whereas mice immunised with a live TCC-containing formulation, were able to resist the virulent infection. Parasitaemia was recorded twice a week until mice from control group succumbed to dead. Animals that were immunised with the formula TCC+rP21 presented a similar parasitaemia to those only immunised with attenuated parasites (Fig. 2B) showing no significant differences at this level. When analysing the parasite load in heart and skeletal muscle, the parasite burden of TCC and TCC+rP21 groups, remained below the levels detected for control groups. Surprisingly, rP21-immunised animals presented an elevated *T. cruzi* parasite load in these organs (Fig. 2C-D). These results could be attributed to the fact that some rP21 protein residues could still be available in the host during the challenge, favoring virulent parasites to invade cells and multiple in the host. In the same way, a similar effect could also have happened in the TCC-rP21 group; however, in this case we speculate that the presence of TCC could counter this. We have previously shown through *in vitro* infection assays that when the protein is added at the same time as the parasites, a significant increase in host cell invasion is observed.^13^ Then, we could speculate that something similar could be taking place in relation to the parasite load found in blood, heart and muscle of rP21 immunised/challenged mice. In this case, rP21 would be involved in favoring parasite invasion, rather than acting as an antigen for triggering a humoral response. The effect of combining rP21 together with attenuated parasites (TCC+rP21) did not translate in an improved protection compared to the one conferred by immunisation with attenuated parasites alone. We could determine that the capacity of rP21 to stimulate cell invasion is higher than its capacity to trigger a robust immune response capable of protecting against a virulent challenge as observed by the lack of protection in the immunisation and challenge experiments.

**Fig. 3:**
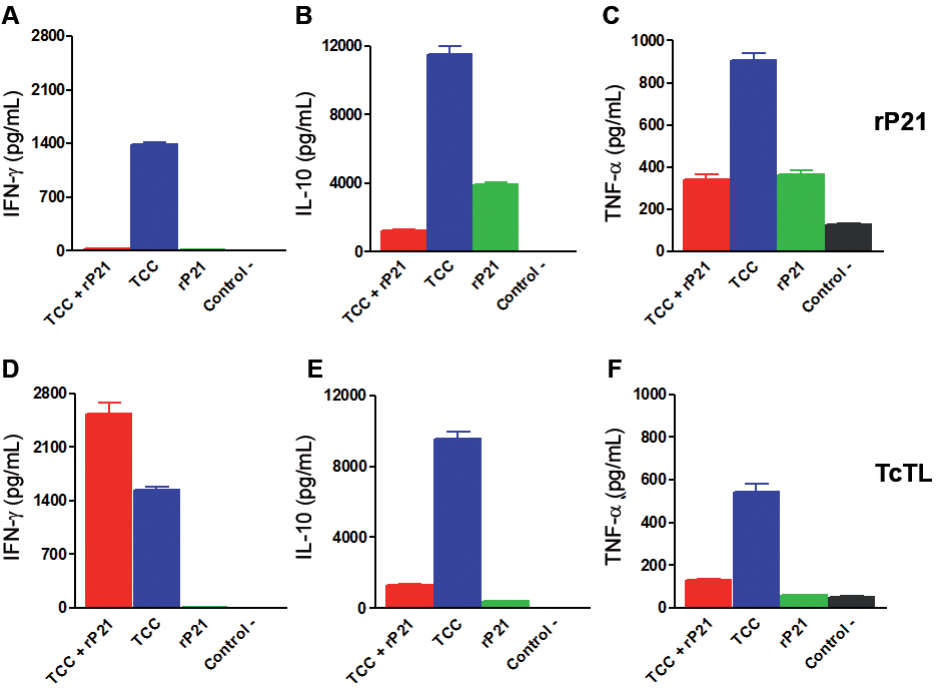
cytokines production by splenocytes of immunised animals. As before, groups of mice (n = 5 per group) were immunised with TCC+rP21, TCC, rP21 and as a negative control, PBS-saponin 5%, which is the vehicle in which all the formulations were resuspended. Four weeks after the last immunisation, two mice were sacrificed, spleens removed and splenocytes collected and stimulated with rP21 or total *Trypanosoma cruzi* lysate (TcTL) to measure by enzyme-linked immunosorbent assay (ELISA), the specific cytokines secretion in the culture medium.

*The cytokine profile evoked by attenuated infection was modified by rP21 addition* - Over the years, an important piece of evidence has been collected indicating that cytokines play key roles in the control of *T. cruzi* acute infection, being IFN-γ, IL-10 and TNF-α among the main cytokines involved.^25^ Further analysis of cytokines production in splenocytes from immunised animals showed that after stimulation with rP21 the levels of the analysed cytokines diminished significantly in the group of mice immunised with the formula TCC+rP21 in comparison with the TCC-group (Fig. 3A-C). When stimulated with total *T. cruzi* lysate a similar pattern was observed (Fig. 3D-F), even more, in this case, an increased in the IFN-γ levels of animals immunised with TCC+rP21 was evidenced (Fig. 3D). These data suggested that saponin-emulsified rP21, when combined with attenuated parasites, is capable of increasing the levels of IFN-g upon stimulus with pathogen’ antigens. Initial IFN-γ production is known to occur early during the course of *T. cruzi* infection and may be important in the development of resistance, being Natural Killer cells the major responsible for its secretion. As a matter of fact, IFN-γ activation during the acute phase of *T. cruzi* infection promotes macrophage activation and reactive oxygen species production, and as a consequence, this higher expression leads to the inhibition of parasite replication.^26^ Indeed recent studies suggests that IFN-g could avoid tolerogenic circuits and reduce parasite burden in heart and skeletal muscle^27^ ^,^ ^28^ and thus favor parasite control in the first steps of infection. Because of these reasons, an up-regulation of IFN-g expression in immunised mice may represent a valuable modulation gained by rP21 supplementation. Still, at the level herein analysed, the increased in the IFN-g production by splenocytes of TCC+rP21 immunised animals failed to promote a better control of parasite replication after challenge. On the other hand, the presence of rP21 diminished the production of TNF-α elicited by the immunisation with attenuated parasites alone, when stimulated with whole parasite homogenate (Fig. 3E-F). High level of TNF-α expression is required for nitric oxide production by the activated macrophages and for achieving parasite early restrain during the first stages of infection. In our experimental approach, the minor production of this cytokines displayed by TCC+rP21 immunised animals, may be seen as an advantage gained by the adjuvant addition, since parasite control was achieved through an IFN-γ-dependent response, avoiding a possible detrimental high expression of TNF-α. There are several works proposing that IL-10 production by T cells favors *T. cruzi* control and prevents the development of a pathologic immune response.^29^ ^,^ ^30^ According to our results, IL-10 seems no to play a fundamental role in controlling parasite replication since the parasite burden was similar in TCC and TCC+rP21 immunised/challenge animals. These data strongly suggest a potential differential modulation of cytokine production by rP21 during the course of infection of attenuated parasites. However, this modulation does not alter or modify the final outcome product of the vaccination.

In summary we described for the first time, the use of a *T. cruzi* recombinant protein as a possible modulator during *T. cruzi* attenuated immunisation. We are allowed to conclude that rP21 possess adjuvant capacity able to modify the cytokine immune profile elicited by infection with attenuated parasites. However, pre-treatment with saponin emulsified-rP21 could lead to exacerbate infection and parasite load in target organs, suggesting the important role that P21 has over the natural invasion process but also pointing out the risks that the administration of this component may have over a second proximal infection. These conclusions may have significant relevance in prophylactic and therapeutic strategies for vaccine development against Chagas disease. What is more, the observations made on *T. cruzi* P21 as an adjuvant component, could be valuable in the future design of immunisation strategies intended for the control of animal reservoirs.
